# Three novel germ-line VHL mutations in Hungarian von Hippel-Lindau patients, including a nonsense mutation in a fifteen-year-old boy with renal cell carcinoma

**DOI:** 10.1186/1471-2350-14-3

**Published:** 2013-01-08

**Authors:** Gergely Losonczy, Ferenc Fazakas, György Pfliegler, István Komáromi, Erzsébet Balázs, Krisztina Pénzes, András Berta

**Affiliations:** 1 Department of Ophthalmology, University of Debrecen, Medical and Health Science Center, 98. Nagyerdei bld, 4012, Debrecen, Hungary; 2 Haemostasis, Thrombosis and Vascular Biology Research Group of the Hungarian Academy of Sciences, Clinical Research Center, University of Debrecen, Debrecen, Hungary; 3 Division of Rare Diseases, Medical and Health Science Center, University of Debrecen, Debrecen, Hungary

**Keywords:** Genotype-phenotype correlation, Germline mutation, Renal cell carcinoma, Von Hippel-Lindau disease

## Abstract

**Background:**

Von Hippel-Lindau disease is an autosomal dominantly inherited highly penetrant tumor syndrome predisposing to retinal and central nervous system hemangioblastomas, renal cell carcinoma and phaeochromocytoma among other less frequent complications.

**Methods:**

Molecular genetic testing of the VHL gene was performed in five unrelated families affetced with type I VHL disease, including seven patients and their available family members.

**Results:**

Molecular genetic investigations detected three novel (c.163 G > T, c.232A > T and c.555C > A causing p.Glu55X, p.Asn78Tyr and p.Tyr185X protein changes, respectively) and two previously described (c.340 + 1 G > A and c.583C > T, resulting in p.Gly114AspfsX6 and p.195GlnX protein changes, respectively) germline point mutations in the VHL gene. Molecular modeling of the VHL-ElonginC-HIF-1alpha complex predicted that the p.Asn78Tyr amino acid exchange remarkably alters the 77-83 loop structure of VHL protein and destabilizes the VHL-HIF-1alpha complex suggesting that the mutation causes type I phenotype and has high risk to associate to renal cell carcinoma. The novel p.55X nonsense mutation associated to bilateral RCC and retinal angioma in a 15-year-old male patient.

**Conclusion:**

We describe the earliest onset renal cell carcinoma in VHL disease reported so far in a 15-year-old boy with a nonsense VHL mutation. Individual tailoring of screening schedule based on molecular genetic status should be considered in order to diagnose serious complications as early as possible. Our observations add to the understanding of genotype-phenotype correlation in VHL disease and can be useful for genetic counseling and follow-up of VHL patients.

## Background

Von Hippel-Lindau (VHL) disease is an autosomal dominantly inherited highly penetrant tumor syndrome affecting 1 in 36,000 individuals worldwide. VHL disease predisposes to retinal and central nervous system hemangioblastomas (HB), renal cell carcinoma (RCC), phaeochromocytoma, pancreatic endocrine tumors, endolymphatic sac tumors among other less frequent complications. Type 1 diseases is accompanied with low risk of phaeochromocytoma, type IIa is associated with high risk of phaeochromocytoma and low risk of renal cell carcinoma, while type IIb is linked to high risk of both phaeochromocytoma and renal cell carcinoma. In type IIc VHL disease only phaeochromocytoma develops. Patients carry a heterozygous germline mutation in the VHL gene. Tumor development is initiated by the somatic inactivation or loss of the remaining wild type VHL allele [1,2]. VHL protein associates with the elongins B and C, cullin2 and Rbx and functions as the substrate recognition component of an E3-ubiquitin ligase that ubiquitinates HIF-1aplha under normoxic conditions resulting in HIF-1alpha proteolysis [3].

The protein model of pVHL19 contains two functional subdomains; the beta domain (residues 63–154 and residues 193–204) and the helical alpha-domain (residues 155–192). Two important binding sites within VHL protein have been identified; one responsible for elongin C binding in the alpha domain (amino acid residues 157–170) and the other in the beta domain responsible for the binding of HIF1alpha (amino acid residues 91–113) [4]. The majority of disease-causing missense VHL gene mutations are located in one of these two binding sites. Molecular genetic defects identified in the background of the disease have greatly helped to understand the role of VHL protein in the hypoxia sensing pathway and to recognize genotype-phenotype correlation, a prerequisite of efficient genetic counseling and patient follow-up [5]. More than 800 different germline mutations are listed in the VHL mutation database ( http://www.umd.be:2020/) [6] and also in a comprehensive analysis based on 945 VHL kindreds [7]. Here, we describe 7 members of 5 families with three novel and one previously reported point mutations in the VHL gene and correlate genetic findings with clinical phenotype.

## Methods

### Patients

Seven members of five unrelated Hungarian families with von Hippel-Lindau disease were investigated at the Division of Rare Diseases and the Department of Ophthalmology, at the University of Debrecen in Debrecen, Hungary between 1998 and 2011. The diagnosis of VHL disease was based on physical and ophthalmological examinations, abdominal CT, craniospinal and abdominal MRI and laboratory tests (routine blood tests and urine catecholamines and vanillylmandelic acid). All examinations were in line with previously reported screening protocols [2,8-10]. One hundred apparently healthy controls were enrolled in the study. Controls, Patients and family members were enrolled after having signed informed consent. All procedures strictly adhered to the Declaration of Helsinki. Approval was obtained from local Institutional Ethics Committees at the University of Debrecen.

### Mutation analysis of the VHL gene

Blood samples from patients and family members were obtained after informed consent. Genomic DNA was purified from peripheral white blood cells. Exons of the VHL gene were amplified using the following primer pairs: 1AF 5^′^-TATAGTGGAAATACAGTAACGAG-3^′^, 1AR 5^′^-GAAGTTGAGCCATACGG-3^′^, 1BF 5^′^- AGAGTACGGCCCTGAAGAA-3^′^, 1BR 5^′^-GCTTACGAGCAGCGTCAC-3^′^, 2 F 5^′^-ATCTCCTGACCTCATGATCC-3^′^, 2R 5^′^- GGGCTTAATTTTTCAAGTGG-3^′^, 3 F 5^′^- TGAGATCCATCAGTAGTACAGG-3^′^, 3R 5^′^- CTAAGGAAGGAACCAGTCC-3^′^. After an initial denaturation step at 95°C for 10 minutes, 40 PCR cycles were performed under the following conditions: denaturation at 95°C for 1 minute, annealing at 56°C for exon 1 and 59°C for exons 2 and 3 for 1 minute and extension at 72°C for 1 min. An additional elongation step for 7 minutes at 72°C followed the final cycle. PCR products purified by ultrafiltration were sequenced by ABIPrism 3100 Genetic Analyzer (Applied Biosystems, Foster City, CA) from both forward and reverse primers. Clinical and genetic data of previously reported VHL cases were obtained from cited publications and from the Universal Mutation Database [6]. Large deletions were tested using the Multiplex Ligation-dependent Probe Amplification (MLPA) VHL kit (MRC-Holland, Amsterdam, NL).

### Evolutionary alignment, SIFT analysis and molecular modeling

Multiple evolutionary protein sequence alignment was performed using the open access source of Constraint-based Multiple Alignment Tool (COBALT) in the case of the p.N78Y mutation [11]. To estimate whether this amino acid substitution affects protein function we also applied the online available SIFT analysis tool ( http://sift.jcvi.org/). SIFT prediction is based on the degree of conservation of amino acid residues in sequence alignments derived from closely related sequences, collected through PSI-BLAST [12]. We were curious whether the SIFT tool could find any difference in the predicted effect between type I and type II missense mutations, therefore we analyzed all germline missense mutations of familial VHL cases reported in the comprehensive analysis of Nordstrom-O’Brien et al. Sixty-six mutations in both type I and II groups were analyzed.

For molecular modeling, starting geometries for the model complexes of VHL-, Elongin C- and HIF-1α proteins [13] were obtained from the RCSB protein data bank (pdb ID: 1LM8). The unresolved N- and C-terminal fragments were substituted by acetyl and N-methyl groups on the available protein fragments then the missing hydrogen atoms were added. Molecular dynamics simulations using periodic boundary condition, explicit TIP3P water [14] molecules, AMBER99SB [15,16] force field and additional Na + and Cl- ions (~0.15 M ionic strength) were carried out with both the wild- and the Asn78Tyr point mutated VHL protein. 4 fs time step and 50 ns total time frame was applied for the constant pressure (10^5^ Pa), constant temperature (310 K), and constant particle number (68612, 68655 and 68548 for the wild and p.Asn78Tyr mutant systems, respectively) simulations. The long range electrostatics forces were calculated by particle mesh Ewald method [17]. The simulations and the analyses of the trajectories were carried out by means of the Gromacs [18] suites of software.

## Results

**Figure 1:**
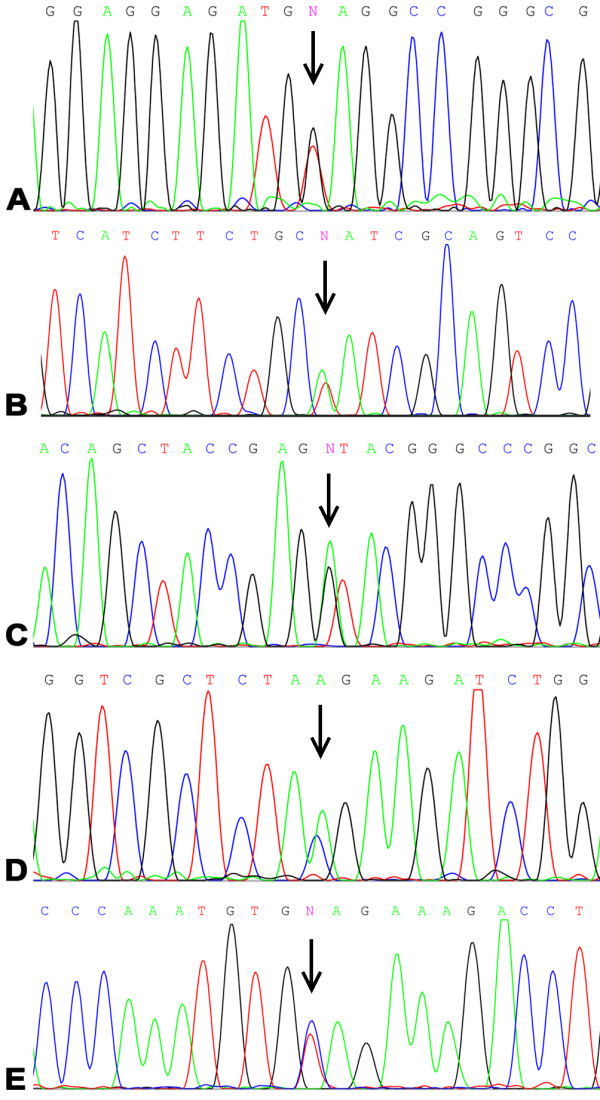
**Electropherograms representing VHL mutations in the study population.** Mutations are indicated by arrows. Panel **A**: c.163 G > T, p.Glu55X; Panel **B**: c.232A > T, p.Asn78Tyr; Panel **C**: c.340 + 1 G > A, p.Gly114AspfsX6; Panel **D**: c.555C > A, p.Tyr185X; Panel **E**: c.583C > T, p.195GlnX.

Molecular genetic analysis of 11 members of 5 unrelated VHL families detected 3 novel and 2 previously described point mutations in the VHL gene. No alterations could be detected in the VHL gene with the MLPA test. All mutations associated to type I VHL disease. None of the mutations could be observed in one hundred control subjects. Phenotype characteristics and genetic data of the study population are summarized in Table 1. Electropherograms of corresponding VHL mutations are demonstrated in Figure 1.

**Table 1:** Summary of clinical and genetic findings

**Family**	**Patients**	**RCC**	**CNS HB**	**Phaeo**	**RA**	**VHL disease type**	**germline mutation**	**localisation**	**novel mutation**	**Predicted protein modification**	**SIFT analysis**
A	IP	15	-	-	15	1	c.163 G > T	exon 1	yes	p.Glu55X	NA
B	IP	-	48	-	48	1	c.232A > T	exon 1	yes	p.Asn78Tyr	damaging
	Brother	-	45	-	45	1	c.232A > T	exon 1	yes	p.Asn78Tyr	damaging
C	IP		14	-	12	1	c.340 + 1 G > A	intron 1-2	no	p.Gly114AspfsX6	NA
	Father	34, bilateral	34	-	38	1	c.340 + 1 G > A	intron 1-2	no	p.Gly114AspfsX6	NA
D	IP	25	25	-	-	1	c.555C > A	exon 3	yes	p.Tyr185X	NA
E	IP	-	41	-	41	1	c.583C > T	exon 3	no	p.195GlnX	NA

Numbers indicate patients’ age at detection of the corresponding VHL tumor. *IP* Index patient, *RCC* Clear cell renal cell carcinoma, CNS *HB* Central nervous system haemangioblastoma, Phaeo: phaeochromocytoma, *RA* Retinal angioma, *NA* Non-applicable.

Family A consisted of two healthy parents and their 15-year-old son, who developed bilateral retinal angioma and serous retinal detachment. In search for VHL manifestations abdominal ultrasound scan detected a mass of 18 mm in diameter in the left kidney. Abdominal MRI scans showed a kidney mass of 15 mm’s in the left kidney and a 10 mm’s diameter mass in the right kidney. After tumor enucleation, histological analysis verified RCC. MRI of the craniospinal axis could not detect any alterations. No deviation from normal values was observed in the laboratory test. The patient was heterozygous for the novel c.163 G > T (p.55GluX) amber nonsense mutation. None of his parents displayed any mutations in the VHL gene indicating a *de novo* mutation.

Two affected probands of family B, sister (index patient, IP) and brother, with a positive family history of VHL disease were examined. The sister developed a retinal angioma of the right eye at the age of 48 years. MRI examination revealed a cerebellar hemangioma in both patients. RCC could not be revealed so far in any of the patients. Molecular genetic analysis detected the novel c.232A > T (p.Asn78Tyr) missense mutation in heterozygous form in both patients.

Family C consisted of two parents and their two daughters. The 12-year-old daughter (IP) was investigated because of a retinal angioma. A spinal cord hemangioma was detected on the craniospinal MRI. No other manifestations of VHL disease have been detected so far in her case. Radiological investigations detected bilateral kidney tumors, a cerebellar and a spinal cord hemangioma in her 34-year-old father. Histological analysis of the kidney verified RCC. Four years later, retinal angioma developed in both of his eyes. The mother and the other daughter were free from any manifestations of the disease. Both affected family members were heterozygous for the c.340 + 1 G > A mutation, located at the first exon-intron junction. The mutation destroys the conserved intronic donor splice site and most likely leads to premature protein termination (p.Gly114AspfsX6). The mother and the healthy daughter showed no mutations in the VHL gene. As in our case, the mutation associated to type I VHL phenotype in a previous report [19].

The 25-year-old male patient (Patient D) was the only available member of the family. His father had died several years ago due to a central nervous system tumor, probably a complication of VHL disease. The patient was diagnosed with cerebellar and spinal cord hemangioma and a subsequently occurring RCC, while other manifestations of VHL disease could not be detected. The novel heterozygous nonsense amber mutation (c.555C > A) was detected in Patient D. The mutation caused a previously reported protein truncation (p.185TyrX) [6,20,21].

Patient E suffered from retinal angioma and cerebellar hemangioblastoma. No other VHL-related manifestations could be revealed so far. Genetic analysis detected the previously described c.583C > T mutation resulting in premature protein termination (p.195GlnX). Interestingly, the mutation has been reported to associate to type I and type II VHL disease as well [20,22,23].

### SIFT analysis and evolutionary sequence alignment

**Figure 2:**
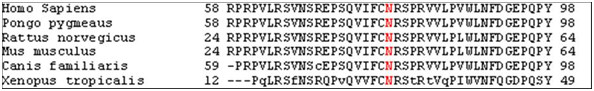
**Multiple sequence alignment of VHL protein.** The Asn78 amino acid is shown in red. Asparagine amino acid in this position is highly conserved among different species.

The majority of missense mutations reported in the literature in familial VHL were predicted to be damaging by SIFT analysis. However, 18% and 35% of the mutations were predicted to be tolerated among mutations associating to familial cases of type I and type II VHL disease, respectively (Fisher’s exact test, p = 0.048). It is to be noted, that the lowest rate (11%) of tolerable mutations was observed in type I VHL disease associating to RCC. As shown by Figure 2, the Asn78 amino acid represents a highly conserved amino acid among different species including Homo Sapiens, Pongo pygmeaus, Rattus norvegicus, Mus musculus, Canis familaris and Xenopus tropicalis. SIFT analysis of the current and previously reported amino acid exchanges at this position also indicated damaging effect on protein function with SIFT scores of 0, 0, 0, 0.03 and 0.04 in case of conversion to Tyrosine, Histidine, Isoleucin, Serine and Threonin, respectively. Median conservation values were below 3.25 in each SIFT analysis indicating high confidence predictions.

### Effect of the Asn78Tyr protein exchange on the molecular structure

**Figure 3:**
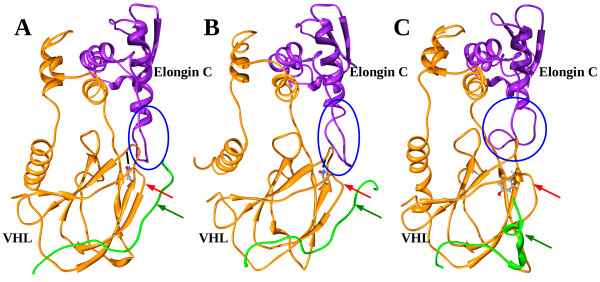
**Ribbon and balls and sticks rendering of a representative snapshot from molecular simulations carried out for model VHL-Elongin C-HIF-1 alpha complexes.** The ribbon model of VHL and Elongin C protein fragments are colored orange and purple, respectively, while the fragment of HIF-1α protein is shown in green. The first and last snapshots from the simulation trajectory of the complex including wild type VHL protein indicates a remarkably stable protein complex (**A,B**). The Asn78Tyr mutation (**C**) remarkably deforms the 77-83 loop structure in the VHL protein. This deformation spreads over the Thr100-Arg107 loop of VHL (marked by red arrow) which finally results in a weakened interaction between this loop and the HIF-1alpha protein (shown by green arrow). The deformed loop structure in VHL deforms substantially the neighboring loop (Arg82-Phe93) structure in Elongin C (shown in blue ellipse in Figure 3B and Figure 3C). Atomtype coloring (C, N, O, H atoms are colored grey, blue, red and white, respectively) was used for the balls and sticks model which were applied for residues which are supposed to play key role in interchain interactions.

Molecular simulation on the model wild type VHL- ElonginC - HIF-1alpha complex shows the protein complex to be remarkably stable as it is demonstrated by the first (Figure 3A) and last (Figure 3B) snapshots from the corresponding trajectory. Whereas comparing the simulation trajectories for the wild type and the Asn78Tyr mutant (Figure 3C) VHL proteins it is clearly demonstrated that this mutation remarkably deforms the 77-83 loop structure in the VHL protein. It can be interpreted by the breaking of the loop-stabilizing H-bond interaction between Asn78 and and Arg82 as well as by a larger space filling property of the tyrosine side chain. This deformation spreads over the Thr100-Arg107 loop of VHL (marked by red arrow) which finally resulted in a weakened interaction between this loop and the HIF-1alpha protein (shown by green arrow). Interestingly, the deformed loop structure in VHL deforms substantially the neighboring loop (Arg82-Phe93) structure in Elongin C (shown in blue ellipse) as well. However, this deformation did not disrupt the interaction between VHL and Elongin C in our simulation.

## Discussion

We have investigated seven VHL patients from five unrelated Hungarian families. After detailed physical, ophthalmological, radiological and laboratory investigations all patients were found to have type 1 VHL disease. Molecular genetic investigations detected three novel and one previously described point mutations in the VHL gene. According to the present and a previous report [24], there are 12 VHL families in Hungary identified so far. Nonsense, missense, frame shift mutations and exon deletions were detected in four, four, two and two families, respectively. These proportions show no major deviation from those of other populations and are comparable to proportions reported by the comprehensive analysis of Nordstrom-O’Brien et al [7]. The novel c.232A > T (p.Asn78Tyr) missense mutation associated to type I VHL phenotype. The site is evolutionary conserved suggesting it is an important amino acid position to maintain protein structure and function. Other mutations at the same codon, namely p.Asn78His, p.Asn78Ser, p.Asn78Thr and p.Asn78Ile associated with type I disease phenotype as well [20,22,23,25-29]. This mutation site is buried within the protein core where mutations were found to associate with lower risk of phaeochromocytoma compared to protein surface missense mutations [23]. N78H, N78I and N78S missense mutations were all reported to coincide with RCC, however the N78T mutation did not show this association [20,22,23,25-29]. According to a later publication RCC associates with high risk to missense mutations located between codon 74 and 90 [30]. The effect of the novel p.Asn78Tyr mutation on VHL protein structure was assessed using molecular modeling. The Asn78 side chain participates in the stabilization of the turn where it can be found. The breaking of the loop-stabilizing H-bond interaction between Asn78 and Arg82 as well as the replacement of this residue with a larger one obviously destabilizes this turn (Figure 3). It can cause partial or global misfolding. The most remarkable effect of this structural reorganization on protein interactions is the high probability of dissociation of the VHL and HIF-1alpha proteins. Despite the turn deformation, disruption of the VHL-Elongin C interaction could not be observed during the molecular dynamics simulation (Figure 3). In vitro analysis of pVHL mutants showed reduced ability to bind and ubiquitinate HIF-1alpha in case of type I, IIa and IIb mutations, on contrary, mutations associated to IIc phenotype showed normal binding and ubiquitination of HIF-1alpha protein. Completely absent HIF-1alpha binding was only observed in consequence of mutations associating to RCC in this study [31]. However, a recent computational study could not confirm the dose-dependent effect of VHL-HIF-1alpha dissociation and suggests that the canonical configuration of the wild-type beta domain is vital for the efficient functioning of the complex and that mutation of any of the residues implicated in the H-bond network in the binding site disrupts HIF binding [32]. Taken together, mutations in the beta domain disrupting the VHL-HIF-1alpha interaction lead to type I disease phenotype with an increased risk of RCC. Based on these data and our molecular modeling results we can conclude that the novel p.Asn78Tyr mutation associates to type I disease and has a high chance to associate to RCC as well, while only minor risk to associate to phaeochromocytoma. In an attempt to assess the effect of all previously reported missense mutations in familial VHL, we applied SIFT analysis and found that in type II disease the rate of tolerable mutations is significantly higher than in type I mutations. This finding further supports that deleterious mutations disrupting protein integrity are more prone to cause type I phenotype. Besides, it suggests that SIFT analysis can be a useful tool when quick prediction of a novel mutation is necessary.

As expected, nonsense mutations associated with type I VHL phenotype. Two of the three nonsense mutations associated to RCC as well. Previous publications show that large germline deletions, nonsense and frameshift mutations associate with type I VHL phenotype [6,20-22,25,33]. Consistent with previous publications indicating that RCC coincides more frequently with nonsense mutations than missense mutations, RCC only associated to MLTP in our patients [23,34]. Nonsense mutations independent of their exonic location associate with earlier age at onset and higher age-related risk of RA and RCC compared to missense mutations and large deletions [23]. Supporting this observation, the patient with the p.55GluX mutation was 15-years-old when bilateral retinal angioma and bilateral RCC was diagnosed, which represents the earliest occurrence of RCC in VHL disease reported so far [1,35]. VHL screening guidelines [2,8-10] recommend a screening schedule for VHL patients regardless of their genetic status in order to recognize VHL complications in a curable stage. The earliest occurrence of RCC was reported in a sixteen-year-old boy. Accordingly, screening guidelines recommend yearly or biannual abdominal MRI from sixteen years of age and yearly abdominal ultrasound starting at 8 years of age. The occurrence of RCC in a fifteen-years old boy with a truncating germline VHL mutation highlights that renal cell carcinoma can be detected in patients with nonsense VHL mutations younger than sixteen years of age. Taken into account that abdominal MRI scans with a higher resolution can detect RCC earlier than ultrasound, patients with nonsense mutations known to associate to RCC and early onset of the VHL disease [23] could be considered for earlier MRI scans. In general, genetic status might be considered to help tailoring individual screening schedules in the future.

## Conclusion

Genotype-phenotype correlation was analyzed and related to the findings reported in previous publications. In our study population we confirmed that the disruption of the VHL protein integrity leading to the disregulation of HIF-1alpha associate to type I phenotype. Accordingly, we also showed that the proportion of non-deleterious mutations is higher in type II than in type I missense mutations among mutations reported in familial VHL cases. Here we show the earliest occurrence of RCC reported in VHL disease so far and suggest individual tailoring of screening schedule based on molecular genetic status. Clinical and genetic data presented in our study can help the routine of genetic counseling and patient follow-up, as well as the presymptomatic molecular genetic diagnostics and contribute to a better understanding of genotype-phenotype correlations.

## Abbreviations

CNS: Central nervous system; MLTP: Mutation leading to protein truncation; Phaeo: Phaeochromocytoma; RA: Retinal angioma; RCC: Renal cell carcinoma; VHL: Von Hippel-Lindau.

## Competing interests

The authors declare that they have no competing interests.

## Authors’ contributions

Acquisition of clinical data: AB, GL, EB, GP. FF and KP performed the molecular genetic analysis. Three dimensional protein modeling was carried out by IK. SIFT and Alignment analysis: GL. Analysis and interpretation of data: AB, IK, FF, GL. Drafting of the manuscript: GL, IK. Revision of the manuscript for important intellectual content: GL, FF, GF, EB, AB. Study supervision: AB. All authors read and approved the final manuscript.

## Pre-publication history

The pre-publication history for this paper can be accessed here:

http://www.biomedcentral.com/1471-2350/14/3/prepub
